# Degradation of Polyacrylate in the Outdoor Agricultural Soil Measured by FTIR-PAS and LIBS

**DOI:** 10.3390/polym10121296

**Published:** 2018-11-22

**Authors:** Dong Liang, Changwen Du, Fei Ma, Yazhen Shen, Ke Wu, Jianmin Zhou

**Affiliations:** 1 The State Key Laboratory of Soil and Sustainable Agriculture, Institute of Soil Science, Chinese Academy of Sciences, Nanjing 210008, China; dliang@issas.ac.cn (D.L.); fma@issas.ac.cn (F.M.); yzshen@issas.ac.cn (Y.S.); kwu@issas.ac.cn (K.W.); jmzhou@issas.ac.cn (J.Z.); 2 University of Chinese Academy of Sciences, Beijing 100049, China

**Keywords:** polyacrylates, soil, degradation, FTIR-PAS, LIBS, PCA

## Abstract

Recently, polyacrylates (PA) have been applied in coated controlled-release fertilizer (CRF), but the impacts of the soil on the degradation of PA have not been evaluated. In this study, an outdoor agriculture soil buried test was carried out for 12 months to investigate the degradation of PA films. The residual degraded films were taken regularly from the soil and analyzed by SEM, Fourier transform infrared photoacoustic spectroscopy (FTIR-PAS) and laser-induced breakdown spectroscopy (LIBS). The concentration of C–H and C=O molecular groups of PA were decreased, and the element concentrations of C, O, K, Si of PA were increased under the degradation process. The surface of PA became rough and the degradation of PA occurred on the surface layer. Principal component analysis (PCA) showed that soil invaded PA. The results indicated that PA were environmentally friendly when applied to CRF. FTIR-PAS and LIBS were advanced in the in-situ surface analysis of the degradation process of the polymer.

## 1. Introduction

Polyacrylates (PA) have been used in numerous products, including coatings, adhesives, and fabrics [1]. In the field of agriculture, PA are used as the coats of controlled-release coated fertilizers (CRFs) [2]. PA adopt waterborne coating technology, which reduces the pollution risk of using organic solvents in production [3]. These polymer materials have good film-forming properties and are relatively cheap [4]. Furthermore, PA coated CRFs increase wheat and maize yields in the North China Plain [5]. When PA are used as soil amendments, they increase the water holding capacity of soils [6] and enhance soil quality and plant growth in heavy metal contaminated soils [7]. PA are reported to be biodegradable in the soil [8], and the main chain degrade in the soil at rates of 0.12–0.24% per six months [9]. White-rot fungus and soil microbes synergistically cooperate in the degradation of PA [10]. There are different definitions for degradation in different fields of science, and the definition that describes the chain scission process during which polymer chains are cleaved to form oligomers or monomers [11] is adapted for this paper. For example, starch is first degraded into the oligomer maltose, and then maltose is degraded into the monomer glucose [12]. However, how the soil environment affects the degradation of PA remains unclear. 

The development of the spectroscopy technology provides the in-situ analysis methods, such as scanning electron microscope(SEM), Fourier transform infrared photoacoustic spectroscopy (FTIR-PAS) and laser-induced breakdown spectroscopy (LIBS), which are suitable to study the surface characteristics of the polymer in the soil. FTIR-PAS and LIBS provide the advantages of rapid and simple operation. These unique features lead to the application of two spectroscopy techniques in a wide range of the samples such as soil samples [13], biological samples [14], polymer samples [15], and more. FTIR-PAS is based on FTIR combined with photoacoustic technology. The highlights of FTIR-PAS include the nondestructive method and the deep scan [16,17]. In this detection process, the modulated infrared (IR) signal is first transformed to a thermal wave, which is then transformed to the photoacoustic signal [18]. FTIR-PAS can be used for the qualitative and quantitative analysis of molecular structure [19], while LIBS focuses on the analysis of elemental composition. LIBS is applied in the characterization of elemental composition. In LIBS, the spectra features of the plasma emission lines related to the elements can be obtained with a laser pulse shot toward the surface of the sample. Using these methods, the degradation process of the polymer can be studied from the molecular structure level to the atom level.

The aim of this work was to study the impacts of outdoor agriculture soil on the degradation of PA and understand the degradation process of PA in the soil. For these purposes, the samples of PA were buried in the agriculture soil for 12 months. The weight loss, surface morphology, the FTIR-PAS and LIBS spectra, and PCA were analyzed.

## 2. Materials and Methods 

### 2.1. Sample Preparation

Waterborne polyacrylate latex was prepared to create the PA film. The thickness and diameter of the PA film were about 2.0 and 10 mm, respectively. The polyacrylate latex was synthesized by the latex polymerization method with Methyl methacrylate (MMA), *n*-butyl acrylate (BA) and methacrylic acid (MAA) as monomers; sodium dodecyl benzene sulfonate (SDBS) and nonyl phenyl polyoxyethylene ether-10 (OP-10) as emulsifiers; and potassium persulfate (KPS) as initiator. The three-necked flask of 1000 mL, mechanical stirrer, reflux condenser, and dropping funnel were used in the polymerization. The aqueous phase was composed by 8.24 g of OP-10, 4.12 g of SBDS and 248 g of water. The organic phase was composed by 110 g of BA, 90 g of MMA and 3.5 g of MAA. The aqueous and organic phases were stirred for 30 min with the swing speed of 550 rpm, and the temperature was raised to 80 °C until the end. The concentration of 25 wt % of the oil–water mixture was used as the initiator solution. The rest of the mixture and the initiator solution (52 mL, 0.01 g/mL, KPS) were fed alternately in four doses over 3 h, and then, the polymerization was conducted under air atmosphere for an additional 3 h. The films were obtained by the casting method. The PA emulsion was cast onto a nonstick substrate, and water was evaporated completely in an oven at 80 °C for 8 h. The basic properties of synthesized polymers were: particle size in emulsion, 100–250 nm; glass-transition temperature (*T*_g_), 6.38 °C; tensile stress at break (MPa), 4.66; Elongation at break (%), 1013; and Young’s modulus (MPa), 36 [20].

The particle size was measured by a dynamic light scattering (DLS) analyzer (Nano ZS90, Malvern Instruments, Malvern, UK). The glass transition temperature (*T*_g_) was measured by the differential scanning calorimetry (DSC, Perkin-ElemrPyris1, Waltham, MA, USA). The mechanical properties of the films were obtained using a universal testing machine (CMT 5254, Shenzhen SANS Testing Machine Co., Ltd., Shenzhen, China) according to procedures outlined in ASTM D 638–03.

### 2.2. Outdoor Soil Burial Test

The outdoor soil burial test was performed in a natural environment. Field trials were conducted between March 2017 and March 2018 at Tangquan Experimental Station of The Institute of Soil Science (32°04′ N latitude, 118°28′ E longitude, and 7 m elevation) in Jiangsu province, China. The soil had been previously used to grow wheat. The basic physicochemical properties of the soil were: pH (H_2_O) 6.7, soil organic matter 24.65 g kg^−1^, total nitrogen 1.51 g kg^−1^, total phosphorus 0.87 g kg^−1^, and total potassium 14.2 g kg^−1^ [21]. The weather information was measured by a WatchDog 2900ET weather station (Spectrum Technologies, Springfield, Illinois, USA). The average temperature was 16.2 °C and the total rainfall was 1118.3 mm in 12 months. The average solar radiation was 161.2 wat m^−2^ and the average wind speed was 2.61 km h^−1^. 

Sixteen films were placed at the burial depth, 10 cm from the soil surface. The samples were separated by 10 cm horizontally. Every 4 films (about 800 mg) were removed after every three months, brushed softly, washed with distilled water several times and dried until they did not lose weight for one month. Drying and weighting were carried out in a constant temperature incubator, set to 25 °C. The air humidity was 22%RH. Finally, the films were submitted for further analysis.

### 2.3. SEM

The evolution of the surface morphologies was monitored with a S-3400N scanning electron microscope (Hitachi, Tokyo, Japan) using 5 kV as the operating voltage.

### 2.4. FTIR-PAS 

The films were scanned directly by FTIR-PAS using the Nicolet 6700 FTIR spectrometer (Thermo Scientific, Waltham, MA, USA) with a Model 300 photoacoustic cell (MTEC Photoacoustics, lnc., Oakland, California, USA). Firstly, the film was put in the cell cup. Secondly, dry helium was released into the cell for 15 s with the speed of 10 mL min^−1^. Finally, the films were scanned 32 times at a resolution of 4 cm^−1^, and the average spectra were obtained. The wavenumber range was 500–4000 cm^−1^, and the mirror velocity was 0.32. The scanning depth of the sample can be calculated as follows:

(1) $$\mu =\sqrt{D/\pi v\gamma }$$

where *μ* represents the thermal diffusion length (μm), *D* represents the thermal diffusivity, *v* represents the moving mirror velocity, and γ represents the wavenumber. For the polymer, *D* is approximately 0.01×10^−5^ m^2^ s^−1^ [22].

### 2.5. LIBS 

LIBS spectra were acquired using a MobiLIBS system (IVEA, Paris, France). The instrumentation consisted of the pulsed Nd:YAG laser (Quantel, Lannion, France), the Mechelle 5000 Echelle spectrometer (Andor Technology, Ltd., Belfast, Northern Ireland), and the intensified charge coupled device camera (iStar, Andor Technology, Ltd., Belfast, Northern lreland). The spectral region was 200–1000 nm. Every film was shot 25 times according to the 5 × 5 matrices, and the average spectra were obtained. The distance between two points in the matrices was 0.5 mm. 

### 2.6. PCA

MATLAB R2016a software (Natick, MA, USA) was used for the spectra data analysis. Firstly, it treated the intensity peak positions in the spectra of FTIR-PAS and LBIS as vectors. Secondly, it formed linear combinations of the vectors by assigning a weight to each vector. 

## 3. Results and Discussion

### 3.1. Weight Loss and Morphological Properties

**Figure 1:**
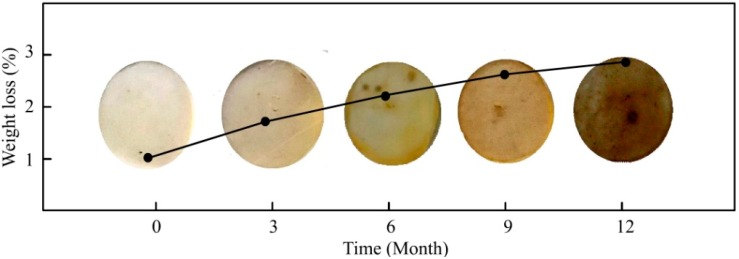
Weight loss and color change of PA films.

The weight loss and color change of the films exposed to the outdoor soil were plotted to evaluate the degradation of PA (Figure 1). It was clear that the was longer the exposure time in the soil, the darker was the color of the samples. The original color of PA was white and would remain so when exposed in water or air. The films lost 1.77% of their weight after 12 months, indicating continued degradation in the soil. During the degradation process, the color depth of the film may be interrelated to the weight loss, and the dark color of the film was resulted from the bond with soil. 

**Figure 2:**
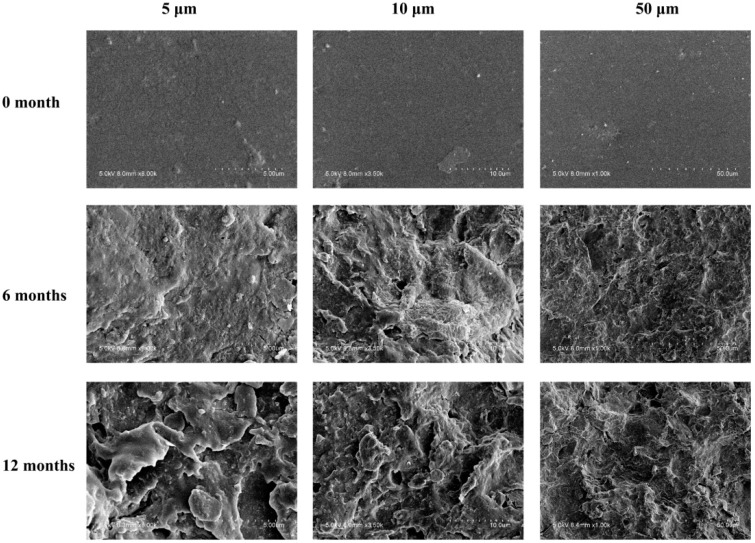
Surface morphologies of PA films before and after buried in soil.

The surface morphologies of PA during burying in outdoor soil were investigated by SEM (shown in Figure 2). The surface of the original PA samples were smooth. After six months buried in soil, their surfaces became rough, and many cracks and holes formed. This was exacerbated after 12 months. This indicated that the PA films had been partially degraded at its surface layer in the soil. PA was reported to be biodegradable [8]. The white-rot fungus, *Phanerochaete chrysosporium*, has been found to biodegrade PA in the soil [10]. By the measurement of ^13^C-labeled PA, main-chain scission occurred primarily at the surface of the polymer when degraded in the soil, i.e., involving “loss ends” and/or oligomers [9].

### 3.2. FTIR-PAS Spectra

**Figure 3:**
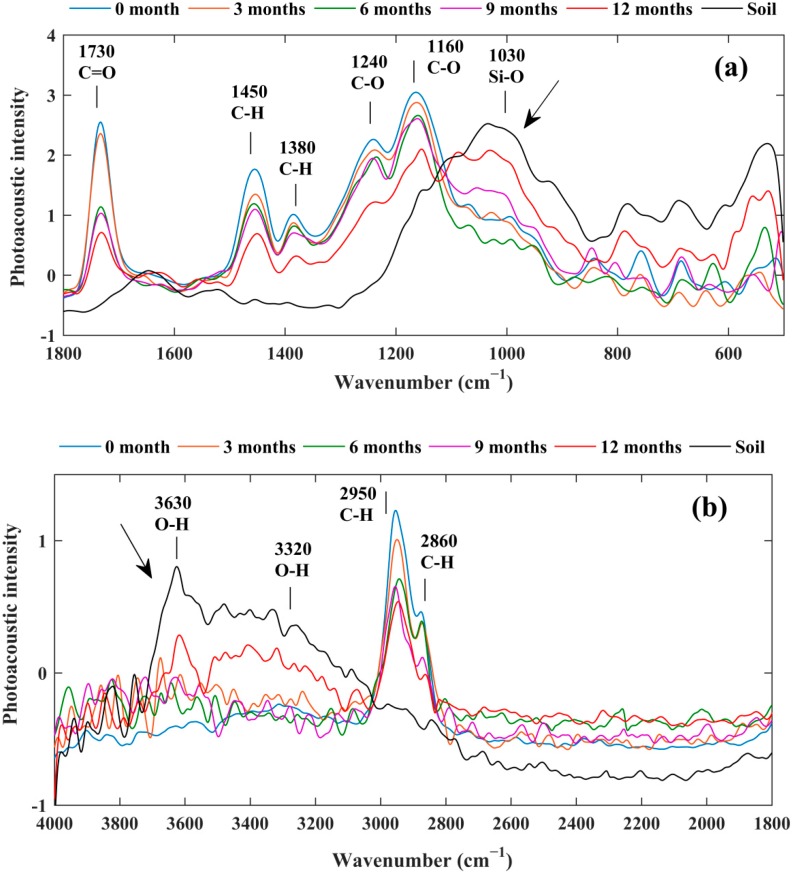
Average FTIR-PAS spectra of PA films and soil in the wavenumber ranges of 500–1800 cm^−1^ (**a**) and 1800–4000 cm^−1^ (**b**).

The chemical structure changes on the surface of the PA films were investigated by FTIR-PAS. Figure 3a,b shows the average spectra in the wavenumber range of 500–1800 and 1800–4000 cm^−1^, respectively. According to Equation (1), the thermal diffusion lengths were calculated (Table 1). On the one hand, the peak intensity of the molecular bonds about C–H (2950 cm^−1^), C=O (1730 cm^−1^), and C–O (1160 cm^−1^), were all on the same order of 0-month film > 3-month film > 6-month films > 9-month film > 12-month film, which indicated that being buried in the soil reduced the intensities of these molecular groups; as PA were mainly formed by these molecular groups, they were highly degraded in the soil. On the other hand, the O–H (3320 cm^−1^) and Si–O (1030 cm^−1^) groups, belonging to the soil, were clearly detected in PA samples after 9 and 12 months. It indicated that soil adhered to the surface of PA, and longer the samples were exposed, more the soil was adhered to PA. When the main-chain scission occurred at the surface and the cracks and holes formed, the surface density of the polymer decreased. Therefore, the molecular composition at the surface of the polymer decreased, and the C=O, C–H and C–O bands decreased. The surface layer of the polymer degraded, but the inside layer of the polymer did not degrade for 12 months. The degradation occurred from the outside layer to the inside layer. This was why the weight loss was small but the molecular bonds decreased relatively more.

### 3.3. LIBS Spectra

**Figure 4:**
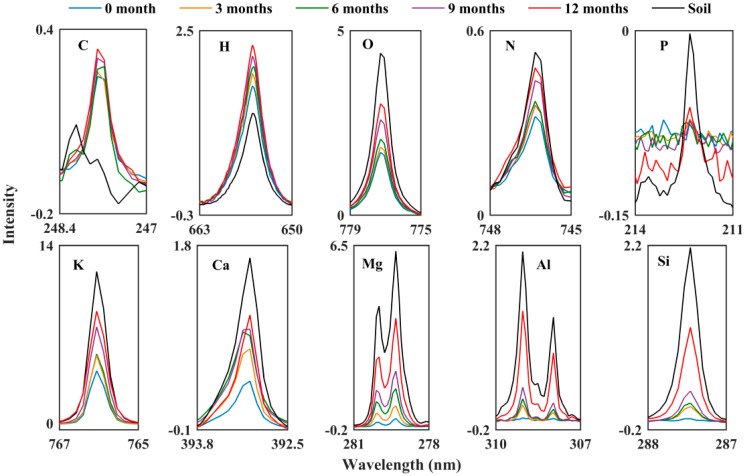
Average LIBS spectra of PA films and soil.

The atomic composition changes on the surface of the PA films were investigated by LIBS. Figure 4 shows the average LIBS spectra of PA films from 0 to 12 months and soil at the selected wavelength ranges from 200 to 1000 nm. The concentration of the nutrient elements, including N (646.5 nm), P (212.3 nm) and K (766.3 nm); the main compositional elements of PA, such as C (589.1 nm), H (656.3 nm) and O (757.5nm); and mineral elements, such as Ca (393.1 nm), Mg (279.4 nm), Al (309.1 nm, 308 nm), and Si (288.1 nm), all followed the order of: 12-month film > 9-month film > 6-month film > 3-month film > 0-month film. It was clear that the mineral and nutrient elements of the soil invaded in the films, and PA received more soil elements the longer they stayed in the soil. According to FTIR-PAS spectra, the PA organic compounds decreased, therefore the increased PA compositional elements were inorganic compounds. The concentrations of most PA films elements were less than soil-forming elements except C and H, because these are the main PA constituents. The adsorption of nutrient elements onto PA samples reduced the nutrients loss in the soil. 

### 3.4. PCA of FTIR-PAS and LIBS

**Figure 5:**
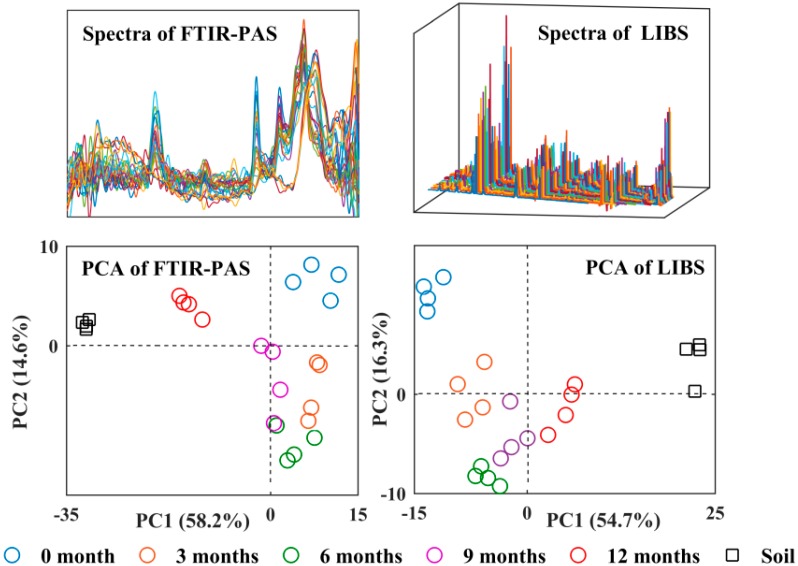
Spectra and PCA scatter plots of FTIR-PAS and LIBS for PA films and soil.

**Figure 6:**
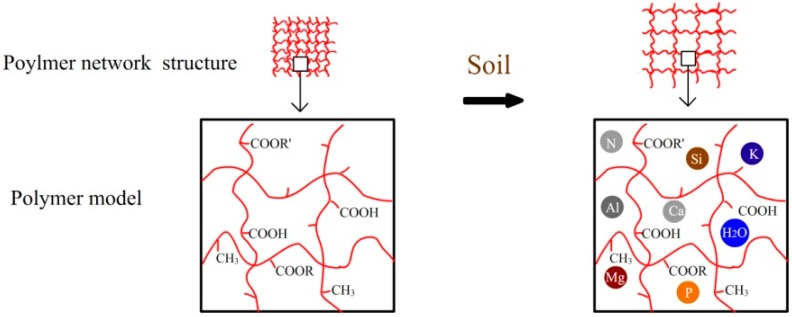
Schematic diagram of PA film buried in outdoor soil.

The differences between the original FTIR-PAS and LIBS spectra (Figure 5) were difficult to identify and analyze due to their similarities and overlapping portions. However, the PCA scatter plots evaluated the spectra explaining the differences among the films. Figure 6 shows the first and second principal components (PC1 and PC2, respectively) at the total wavenumber ranges of 500–4000 cm^−1^ in the FTIR-PAS spectra (72.8% total variance) and the total wavelength of 200–1000 nm in the LIBS spectra (71.0% total variance). Both PCA scatter plots of FTIR-PAS and LIBS spectra followed the same phenomenon: the longest distance in PC1 was between the 0-month films and soils. Additionally, the longer the PA films remained in the soil, the longer distance between 0-month samples and the shorter distance between the soil samples. This illustrated that, once the PA samples were buried in the soil, the two kinds of PA spectra would become more similar to the soil over time, due to the interaction between PA and the soil, as well as soil adhered to the surface of PA.

### 3.5. The Degradation Process of PA in Soil

The degradation of PA in the soil was a combination of various degradation pathways, because the soil environment was very complicated. The factors of the soil such as pH, temperature, humidity, and microorganism all had impacts on the degradation of the polymer. These factors were always changed in the different seasons of one year. Except the biodegradation in the soil, the uptake of water was also important in the degradation. The hydrophilic groups (–COOR and –COOH) of PA provide it with hydrophilic performance. The swelling degree of PA increased to 35% within fourteen days when put it into water [3]. The soil humidity was not stable, and therefore the swelling degree of the film was not stable either. The inorganic and organic groups in the soil were absorbed on the surface of PA(shown in Figure 6). When the PA films were put in the soil, they underwent the repetitive process of adsorption and evaporation because of rainfall and irrigation. Accordingly, the films continuously expanded and contracted, allowing inorganic and organic components of the soil invaded the film by surface contact, causing the surface to become rough. Furthermore, the chain scission process occurred, allowing oligomers to leave the surface of the PA film, decreasing the surface density. This process may have been influenced by the agriculture soil environment, due to hydrolysis, biodegradation, photo degradation, wind erosion and dissolution [23]. The soil elements absorbed in the surface could increase the weight of the film. Therefore, the actual weight loss was in excess of 1.77% for twelve months. However, the soil elements absorbed in the surface layer of the film was thin and the their weight could be ignored. Additionally, PA formed an interpenetrating network [24], as seen in FTIR-PAS spectra, which showed that polymer network structure of PA became loose over burial time. LIBS spectra indicated that the network structure could incorporate and blend with a larger number of inorganic groups, such as nutrient and metallic elements. In the end, PA may eventually become the components of the soil.

## 4. Conclusions

This study shows that PA were clearly degraded in outdoor agriculture soil, and soil adhered to the surface of the PA. The concentration of the mineral and nutrient elements of the PA samples increased over time. FTIR-PAS and LIBS spectra of PA became more similar to the soil as burial time increased. The surface morphology study further confirmed the spectral results; degradation of PA occurred on the surface. This polymer could be widely applied in the coat of CRFs. The combination of FTIR-FAS and LIBS provided the structure and component information of the polymer, which indicated great potential for the understanding of the interaction between the environment and the polymers in the degradation process.

## Figures and Tables

**Table 1:** Assignments of main absorbance bands at different thermal diffusion depth.

Assignment	Wavenumber (cm^−1^)	Thermal Diffusion Length (μm)
O–H stretching (–OH)	3320	5.48
C–H stretching (–CH_3_) C–H stretching (–CH_2_ )	2950 2860	5.81 5.90
C=O stretching (–COOR)	1730	7.58
C–H deformation (–CH_3_, –CH_2_–)	1450	8.28
C–O–C stretching (–COOR)	1160	9.26
Si–O–Si stretching (–SiO–)	1030	9.82
